# Yawning as Therapy? The Potential of the Conditioned Yawn Reflex as a Novel Treatment for Insomnia Disorder

**DOI:** 10.1111/jsr.70142

**Published:** 2025-07-23

**Authors:** Colin A. Espie

**Affiliations:** ^1^ Sir Jules Thorn Sleep & Circadian Neuroscience Institute, Nuffield Department of Clinical Neurosciences University of Oxford Oxford UK

**Keywords:** CBT, insomnia, treatment, yawning

## Abstract

In 1986, Provine, the pioneer of yawning research wrote that ‘Yawning may have the dubious distinction of being the least understood, common human behaviour’ (p. 120); and so yawning remains some 40 years later, as something of a biological and social curiosity. However, this article examines contemporary scientific understanding of this age‐old conundrum, proposing not only that yawning is a universal component of sleep's normal stimulus control paradigm, but that the conditioned yawn reflex might be harnessed to treat insomnia disorder. The core features of yawning as a ubiquitous, involuntary, periodic and conditionable behaviour; its associated actions on arousal, biofeedback and selective attention, as well as thermoregulation and airway patency; and its potential to signal and promote sleep engagement, lead to the proposition that the conditioned yawn reflex as therapy (CYRaT) is a feasible and potentially effective novel therapeutic for sleep‐onset and sleep‐maintenance insomnia disorder. Much research is required to test this hypothesis, but the article describes preliminary protocols for the administration and testing of CYRaT that might be utilised for this purpose.

## 1 Introduction to Yawning

The title of this article reflects a grant award that my colleagues and I received over 20 years ago (Espie, C.A., Anderson J., Russell, A., & Douglas, N.J: The Wellcome Trust [Showcase Award Scheme, 2002: ref. 070969/Z/03/Z]). In part, I am now attempting to assuage my guilt in not having progressed this line of research beyond a couple of abstracts (Reid et al. 2004, 2005). However, I also want to share a lingering thought that ‘yawning as therapy’ still contains the kernel of a good idea. Consequently, I summarise what we know about yawning and its functions and speculate on how we might actively utilise yawning as a novel behavioural therapeutic to address insomnia disorder. My hope is that this will stimulate applied experimental and clinical research on this ubiquitous phenomenon.

### 1.1 What is Yawning?

Yawning is a curious behaviour (R. R. Provine 2012) and a mystery of physiology and disease (Walusinski 2010a, 2010b), yet whatever yawning is it is remarkably conserved. Charles Darwin's notebooks (1836–1844) include his contemplation that yawning was important in natural selection (Barrett et al. 1987, cited in Gallup 2022). From an evolutionary perspective, yawning is ubiquitous among humans, nonhuman primates, mammals, birds and other vertebrates (Smith 1999) and is hard to suppress, being ‘contagious’ in social animals (R. Baenninger 1987; Gallup and Wozny 2022; Valdivieso‐Cortadella et al. 2023). Yawning is regarded as ‘*halfway between a reflex and an expressive movement*’ (Barbizet 1958, 203), comprising a fixed or *stereotyped action pattern* (SAP) involving three phases: a long inspiration (4–6 s), a brief peak or acme (2–4 s) and rapid expiration, with a maximum total duration (of around 10 s), accompanied by a coordinated motor pattern including opening of the jaw, closure of the eyes and contraction of the facial muscles, recurring with periodicity of ~1 min (R. R. Provine 1986; Chouard and Bigot‐Massoni 1990; Daquin et al. 2001; Krestel et al. 2018; Gallup 2022). Yawning also presents throughout the life course, being first evident in the foetus at 12–14 weeks' gestational age (Giganti and Salzarulo 2010).

**FIGURE 1:**
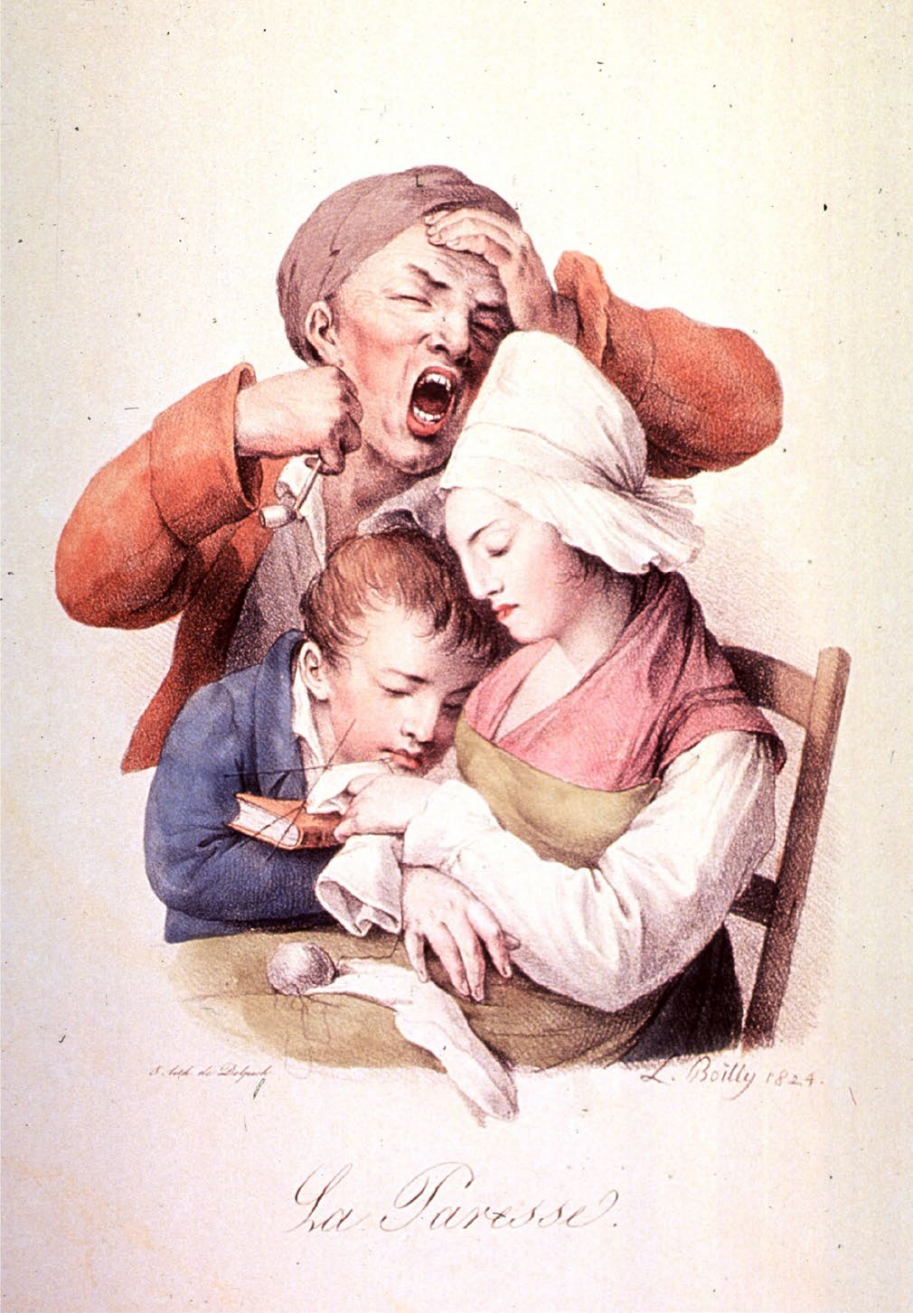
Caricature of sleep or boredom; a woman is sitting in a chair asleep; a boy next to her is asleep also; a man behind them is yawning. Images from the History of Medicine (IHM), Boilly, Louis Léopold, 1761–1845 (artist). Publication: 1824. National Library of Medicine (image in the public domain).

Walusinski (2010a) provides an engaging historical perspective from Hippocrates, who associated yawning with imminent fever (c. 400 bc), through Sennert, Boerhaave and de Gorter who attributed yawning to cerebral anaemia (15th–18th century), to the early 19th century when yawning was thought to presage other pathological states such as gout, ‘excessive evacuation’, serious injury, internal inflammation, hysteria and hypochondria (Landre‐Beuavais 1815). The association of yawning with sleep and sleepiness as well as with boredom and laziness, is illustrated in art (e.g., Figure 1: National Library of Medicine, 1824) and documented in literature, for example, the slothful Oblomov yawned to such an extent that *Oblomovism*, was characterised as an ‘utter inertness resulting from apathy towards everything that goes on in the world’ (Dobrolyubov 1859, 344). More generally, yawning has been regarded as poor etiquette. Rule #5 (of 110) from George Washington's (c. 1814) treatise on decent behaviour was ‘If you cough, sneeze, sigh, or yawn, do it not loud but privately; and speak not in your yawning, but put your handkerchief or hand before your face and turn aside’ (Washington 1888). To some extent yawning continues to be a socially stigmatised behaviour to the present day (Brown et al. 2022).

### 1.2 Why Do We Yawn?

**FIGURE 2:**
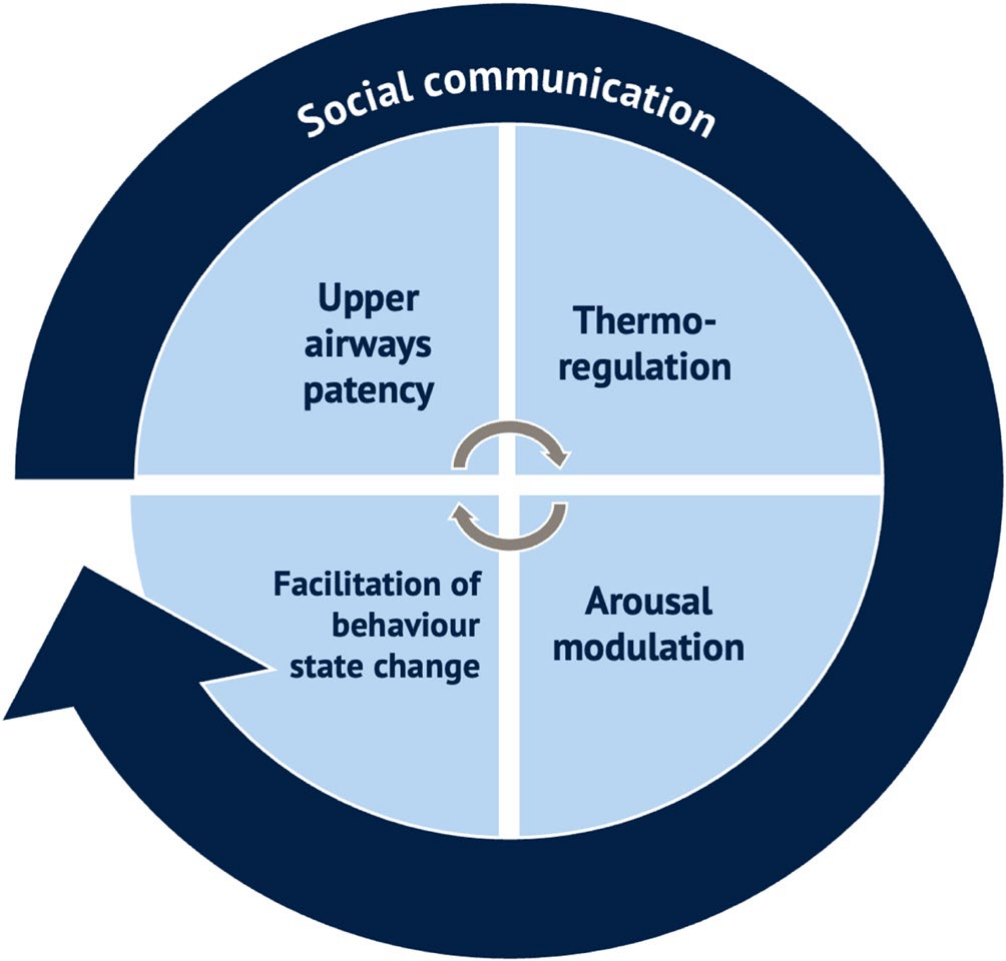
Yawning serves both physiological and social (non‐verbal) communication functions, including biofeedback. These functions likely interact.

Yawning appears to serve important functions, either in physiology or social behaviour (Guggisberg, Mathis, Schnider, and Hess 2010; Gallup 2022), as summarised in Figure 2.

#### 1.2.1 Securing Upper Airway Patency

The traditional respiratory function view, that yawning is facilitated by high levels of carbon dioxide (CO_2_) and/or low levels of oxygen (O_2_) in the blood, has been largely discredited. Provine, Tate, and Geldmacher's (1987) careful experimental work demonstrated that even when CO_2_ was a hundred times greater than the normal concentration level found in ambient air, yawn frequency did not increase, although subjects did dramatically increase their breathing rate and tidal volume. Likewise, breathing 100% O_2_ did not inhibit yawning. Nevertheless, secondary analysis of papers linking obstructive airway conditions to the frequency of yawning suggests that yawning may play a significant physiological role in the repositioning of pharyngeal muscles and widening of the airway lumen, thereby potentially promoting blood oxygenation (Doelman and Rijken 2022). This possibility was first mooted by Hanning (2001). If further primary research were to indicate that yawning does influence airway physiology, then yawning might have beneficial effects, at least in clinical populations if not in general. For example, it has been hypothesised that stimulated yawning might counteract respiratory complications due to Obstructive Sleep Apnoea (OSA), post‐operative lung collapse and difficulty when swallowing. There is also a potential, theoretical application to opioid use, which inhibits yawning (Doelman and Rijken 2022).

#### 1.2.2 Thermoregulation

Second, yawns may be implicated in thermoregulation, triggered by rises in brain temperature and providing compensatory brain cooling (Massen et al. 2014), through increased cerebral blood flow, ventilation of the sinus system and counter‐current heat exchange (Counter‐current heat exchange: a counterflow mechanism that enables fluids at different temperatures flowing in channels in opposite directions to exchange their heat content without mixing [R. Hine 2014; A Dictionary of Biology (8th edition). Oxford University Press]) with ambient air (Gallup and Gallup 2008; Gallup and Eldakar 2013). This is interesting because it is thought that slow‐wave sleep (SWS) in mammals and birds is controlled by thermoregulatory mechanisms and provides brain and body cooling as a homeostatic feedback process (McGinty and Szymusiak 1990). The circadian rhythm of core body temperature is induced by pineal melatonin secretion during the night‐time hours (Cagnacci et al. 1992). Moreover, dissociation of the rapid decline of core body temperature from when we choose to sleep may be associated with insomnia (Lack et al. 2008). In short, sleep is heavily temperature dependent and preparation for sleep may be a thermoregulatory behaviour (Harding et al. 2019; Herberger et al. 2024). There is concomitant interest in temperature‐regulating devices that deliver frontal cerebral brain cooling as a therapy for people with insomnia (e.g., Roth et al. 2018). In parallel, the cortisol hypothesis suggests that rises in cortisol are associated with yawning, and serve to cool the brain (S. B. Thompson 2014; S. B. N. Thompson 2015), with increased electro‐muscular activity observable around the jaw as yawning and cortisol elevation occurs (S. B. N. Thompson 2013; Thompson et al. 2018).

#### 1.2.3 Arousal Modulation

Third, the brain arousal hypothesis suggests that yawning may diffusely activate the brain, increasing arousal particularly in circumstances where the environment provides inadequate stimulation and when the anticipated consequences of low arousal could be perceived as important or hazardous (R. Baenninger 1997). Several groups have conducted controlled experiments to test yawning's effects upon arousal modulation. Temporary increases in autonomic arousal have been demonstrated immediately subsequent to yawning, measurable by increases in heart rate, reactivity in galvanic skin conductance and increased intracerebral blood flow (Greco and Baenninger 1991; Corey et al. 2011; Matikainen and Elo 2008; Provine and Hamernik 1986) Such work suggests that yawning may be associated with an arousing effect both behaviourally and physiologically. However, EEG‐based indices of vigilance have not demonstrated reliable activations. Spectral power across a range of EEG frequency bands either did not significantly change (Laing and Ogilvie 1988) or suggested greater drowsiness rather than arousal increases after yawning (Guggisberg et al. 2007). Where decreases in slow EEG activity and increases in fast activity have been noted, the effects have been notably transient (Regehr et al. 1992), consistent with the autonomic literature. Gallup (2022) suggests that such conflicting findings may be explained by circadian factors because increases in arousal following yawning occur mainly during waking or active states rather than during periods of sleepiness or fatigue before resting or sleep‐onset (Kasuya et al. 2005).

#### 1.2.4 Facilitation of Behaviour State Change

**FIGURE 3:**
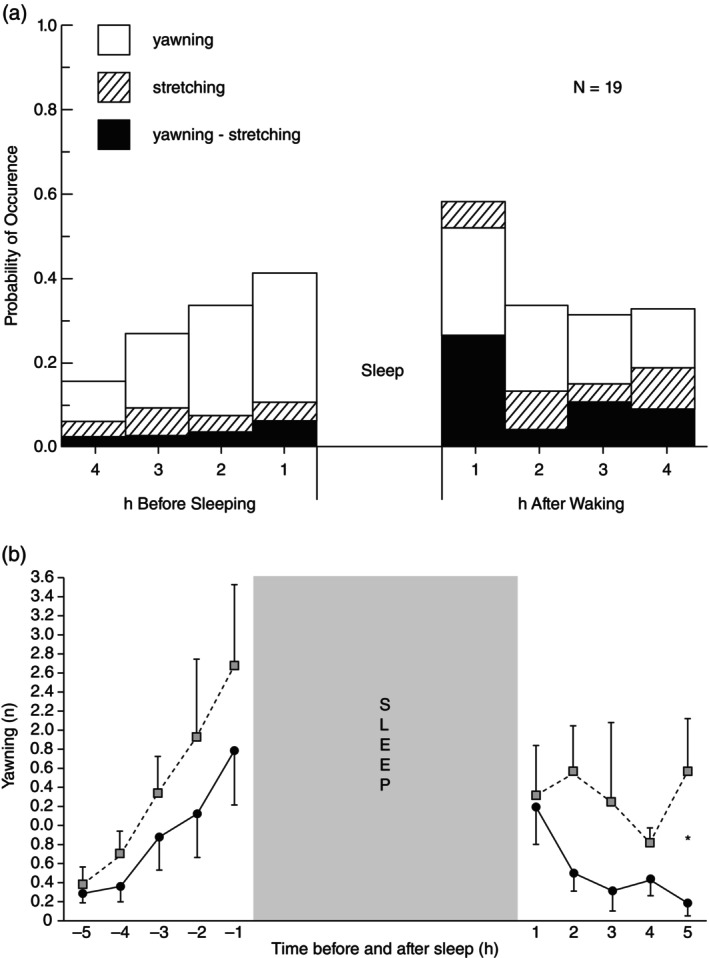
(a) Mean proportion of days on which yawns, stretches, or concurrent yawns and stretches occurred during the 4 h before sleeping and after waking during a 1‐week period. From Provine, Hamernik, and Curchack (1987), reproduced with kind permission. (b) Yawning frequency in evening (■) and morning (●) types before and after sleep (*n* = 16). Data presented as means ± SE, *p ≤ 0.05. From Zilli et al. (2007), reproduced with kind permission.

Fourth, yawning may facilitate what has become known as behaviour state change (R. R. Provine 1986, 2005). Neonates yawn within 5 min of birth (Walusinski 2006) and behavioural and EEG recordings before and after spontaneous yawning indicate that yawning in adults occurs during states of low vigilance, substantiating the notion that it is provoked by sleepiness (Guggisberg, Mathis, and Hess 2010). Yawning occurs most frequently in the hour preceding bedtime and in the hour after rising; often accompanied by stretching primarily in the morning (Figure 3a; Provine, Hamernik, and Curchack 1987). Both morning‐ and evening‐types yawn in the lead up to sleep‐onset but differ after awakening when evening‐types have higher and stable yawning whereas morning‐types show a decline (Figure 3b; Zilli et al. 2007). Moreover, modest peaks also occur late morning and mid‐afternoon, consistent with circadian dips. The state change hypothesis that ‘yawning is a response to and a facilitator of change in behavioral or physiological state’ (R. R. Provine 2012, 37) suggests that yawning may stave off drowsiness, whereas yawning and stretching engage wakefulness.

Importantly, whether or not yawning directly impacts arousal, people are aware of its association with gradients in sleep and wake functioning. This perspective is commonly understood even in lay terms. A brief historical digression (see Table 1) demonstrates that yawning has for long had popular appeal, as well as being seen as potentially therapeutic.

**TABLE 1:** Popular perspectives on yawning in the late 19th century.

Yawning as a therapeutic	Yawning as social contagion
In *A Plea for Yawning* (1893) Dr. N Naegell advises that yawning is one of ‘*nature's remedies’* advising people ‘*… not to concern themselves with squeamish politeness, but every morning and evening … to exercise the lungs and all the muscles of respiration by yawning and stretching*’ He also notes that such ‘*(such)sensations are agreeable*’ and are associated with a ‘*feeling of comfort*’ The Youth's Companion (1827–1929) Feb 9, 1893: 66, 6: Proquest p. 79	Referring to an ‘*inveterate delinquent*’ who was about to be apprehended in a Paris theatre the newspaper reports: ‘*Ere'long, however, he began to yawn, and soon the two policemen took to yawning in sympathy. Their neighbours unconsciously followed suit, then contagion spread … The pit, boxes and gallery were yawning as they never had before. Even the actors … could not resist the example set them*’ The contagion of yawning. The Washington Post (1877–1922); Feb 5, 1888; ProQuest Historical Newspapers: The Washington Post p. 4 (Washington 1888)
Attributed to a French Physician ‘*Not only is it very healthy to yawn, but artificial yawning should be resorted to in cases of sore throat, buzzing of the ears, catarrh, and like troubles*’ Chicago Daily Tribune (1872–1922); Oct 6, 1895. ProQuest Historical Newspapers: Chicago Tribune, p. 40	‘*The tendency to imitate yawning is akin to one other spasmodic action of the breathing apparatus, laughing. Whoever sees a laughing (yawning) picture will engage in it by sympathy*’ Yawning—What is it's causes, and why is it catching? *American Phrenological Journal* (1838–1869); Oct 1869: 49, 10: ProQuest p. 397

*Note*: Materials sourced and shared by Dr. A. Roger Ekirch, with thanks.

#### 1.2.5 Social Communication

Finally in Figure 2, yawning has been described as a ‘paralinguistic signal for drowsiness’ (Provine, Hamernik, and Curchack 1987, 152). In Figure 2, I suggest that this social communication function is best understood as a ‘wrapper’ around the other hypotheses, at least in terms of the potential role of yawning in behavioural therapy. This is first because a *spontaneous yawn* communicates contemporaneous biofeedback to the individual that his/her alertness is low, leading to a personal behavioural activation response (as per the behaviour state hypothesis); and second, simply observing yawns communicates a *contagious yawning* response which synchronises group behaviour (e.g., Sauer and Sauer 1967; Massen et al. 2012; Gallup 2022). This social phenomenon is widely observed in primates and in some other mammals as well as in humans (e.g., Anderson 2010; Norscia and Palagi 2011; Norscia et al. 2021; Casetta et al. 2021; Gallup and Wozny 2022; Walusinski 2022). Contagious yawning is an *echophenomenon*, like the automatic imitation of another's words (echolalia) or actions (echopraxia), the neural basis for which may be linked to disinhibition of the *mirror neuron system* (MNS) and hyper‐excitability of the cortical motor area (Ganos et al. 2012; Schürmann et al. 2005). fMRI studies suggest that the MNS is activated by visually perceived yawning, implying that contagious yawning is based on a functional substrate of empathy and social interaction (Haker et al. 2013), and that yawn contagiousness may be related to increased activation in the amygdala and cingulate cortex (Platek et al. 2005; Schürmann et al. 2005). Yawning is involuntarily elicited as a SAP by seeing others yawn (including on videotape), reading about yawning, or even thinking about yawning (e.g., R. R. Provine 1986, 1989; Arnott et al. 2009; Walusinski 2022). Indeed, it is difficult to stifle contagious yawns, and direct attempts to resist generally increase the urge to yawn (Brown et al. 2017). After noting the lack of empirical evidence for a functional interplay between yawning and vigilance, Guggisberg, Mathis, and Hess (2010) highlight by way of contrast the ‘relatively solid evidence … for social yawns’ and suggest that ‘*the regulating function of yawning might not take place in individuals but rather be effective in social groups. Thus, yawning may be a non‐verbal form of communication that helps synchronize behavior within groups*’ (p. 52).

## 2 Yawning in Clinical and Applied Contexts

It is important next to consider the part that yawning and its measurement play in our understanding of performance, and of health conditions in general, as well as of sleep disorders in particular.

### 2.1 How Does Yawning Relate to Human Performance and Its Measurement?

Most of the literature has focused on driver vigilance and the associated risk of road traffic accidents, with drowsy driving accounting for 25% of fatal accidents (RSPA 2011). A variety of fatigue detection systems incorporate real‐time appraisal methods and associated software algorithms (Akrout and Mahdi 2016; Gaidar and Yakimov 2019; Anderson et al. 2013; Kassem et al. 2020; Cori et al. 2023; Mohd Noor and Ibrahim 2010). Systems that detect excessive blinking, percent of eyelid closure (PERCLOS) and yawning events, have been of interest; and because driver fatigue is characterised by high yawning frequency, mouth opening, mouth edge and lip detection have been explored using both video and thermal images (e.g., Alioua et al. 2011; Safarov et al. 2023; Knapik and Cyganek 2019; Yang et al. 2021). Mouth covering during yawning has also been studied (Jie et al. 2018). Albadawi et al. (2022) provide a helpful summary of detection systems. Surprisingly, there remains conflicting advice about yawning. The industry leader Optalert (2024) suggests that: ‘*many people yawn while driving on an open stretch of road and understimulated*. *This is not a safety risk. On the contrary, it is a sign that they are actively maintaining vigilance*’; whereas the National Safety Council's campaign for Drowsy Driving Prevention Week (NSC 2024) refers to ‘frequent yawning or difficulty keeping your eyes open’ as a key indicator of being ‘too tired to drive’.

Fatigue is also a readily identifiable and potentially preventable cause of aircraft accidents (Dawson et al. 2017) and may be identified through symptoms that include yawning, slowed blinking, the presence of a headache, eye‐rubbing, head droops and microsleeps (Australian Civil Aviation Safety Authority 2012; Keller et al. 2022). However, prominent studies on pilot fatigue appear not to have specifically measured or reported on yawning (e.g., Bendak and Rashid 2020; Berberich and Leitner 2017; Mannawaduge et al. 2024; Naeeri et al. 2019; Dinges et al. 2005). Although PERCLOS and yawn frequency appraised from video recordings have contributed to the accuracy of fatigue detection in a recent study of air traffic controllers (Huang et al. 2024).

Likewise, yawning is not referred to in standard self‐report measures of fatigue. The Multidimensional Fatigue Inventory (Smets et al. 1995), Fatigue Severity Scale (Krupp et al. 1989) and Fatigue Assessment Scale (Michielsen et al. 2003) do not mention yawning, although the 30‐item Fatigue Assessment Scale for Construction Workers has an item on ‘yawning’ which loads on the mental rather than the physical fatigue subscale (Zhang et al. 2015).

### 2.2 How Does Yawning Relate to Clinical Populations?

From a clinical perspective, yawning has been studied mainly where it is pathological, usually in association with CNS disorder. For example, spontaneous yawning is a frequent symptom in neurological conditions such as Parkinson's disease, stroke and epilepsy and presents more rarely in patients with intracranial hypertension, brain tumour, migraine, multiple sclerosis, amyotrophic lateral sclerosis, and hysteria (Krestel et al. 2018; Teive et al. 2018). Yawning also occurs in encephalitis, renal insufficiency and hypotension (Cattaneo et al. 2006; Khungar and Poordad 2012; Cronin 1988). It is noteworthy that whereas the contagious effect of yawning may be impaired in people with theory of mind/autism spectrum disorder (ASD), which affects social and communicative development including empathy (Senju et al. 2007), spontaneous yawn production and daily distribution are not (Giganti and Esposito Ziello 2009). Indeed, problems with empathy explain only very small amounts of variance in susceptibility (Massen and Gallup 2017; Franzen et al. 2018). Yawning is also observed in psychiatric conditions, particularly in anxiety disorder (Daquin et al. 2001; Walusinski 2009). Indeed, experimental studies on rats have demonstrated that classical fear conditioning induces yawning behaviour, suggesting that a neural pathway from the central nucleus of the amygdala to the hypothalamic paraventricular nucleus may be involved in the induction of emotion‐induced yawning behaviour (Kubota et al. 2023).

Attention has been paid to the behavioural pharmacology of yawning (see Collins and Eguibar 2010 and Patatanian and Williams 2011 for overview). In brief, yawning may be influenced by a complex set of neurotransmitter mechanisms including excitatory cholinergic, peptidergic and serotonergic influences, together with dopaminergic and noradrenergic inhibitory influences and their interactions. Dopamine agonists such as apomorphine may increase yawning, particularly in afternoon doses (Blin et al. 1990; Lal et al. 2000), and there are reports of yawning as a side‐effect of SSRI treatment, usually not accompanied by sleepiness (Gutiérrez‐Álvarez 2007; Roncero et al. 2013; Hensch et al. 2015; Beale and Murphree 2000). Yawning side‐effects are rarely serious and typically dissipate when the drug concerned is withdrawn. Yawning may also be associated with opiate withdrawal (Substance Abuse and Mental Health Services Administration (US) 2006), and with caffeine withdrawal (Sajadi‐Ernazarova and Hamilton 2023).

### 2.3 How Does Yawning Relate to Sleep Disorders?

The Psychomotor Vigilance Task (PVT: Wilkinson and Houghton 1982; Dinges and Powell 1985) is the gold standard measure of human vigilance in experimental sleep restriction and sleep deprivation research (Van Dongen and Dinges 2005; Hudson et al. 2020). Yet despite its common association with sleepiness, I could not find reference to yawning across the extensive PVT literature. Hans Van Dongen (Washington State University, USA) responded to my query on this matter:

We do not systematically record yawning events in our experiments, but like most everyone else we do use observations of yawning informally, as one of the symptoms indicating that participants are struggling to stay awake and need research assistant attention to help them stay awake (personal communication, November 27, 2024).

Even in the case of narcolepsy and idiopathic hypersomnia, in which excessive daytime sleepiness (EDS) is a cardinal symptom, diagnostic criteria (e.g., ICSD‐3) and highly regarded textbooks (e.g., Overeem et al. 2001; Dauvilliers et al. 2007) and narcolepsy measurement scales (Swiss Narcolepsy Scale: Bargiotas et al. 2019; Narcolepsy Severity Scale: Dauvilliers et al. 2020) do not even refer to yawning as a symptom. This stands in contrast with publicly accessible sites which suggest that excessive yawning may be caused by sleep deprivation resulting from narcolepsy, as well as by OSA (e.g., https://www.sleepfoundation.org/physical‐health/excessive‐yawning#causes‐of‐excessive‐yawning, accessed 16 June 2024). Moreover, of the Epworth Sleepiness Scale (ESS: Johns 1991), Stanford Sleepiness Scale (Hoddes et al. 1973) and Karolinska Sleepiness Scale (KSS: Akerstedt and Gillberg 1990), only the KSS has an item ‘extremely sleepy–fighting sleep’ that may implicitly include yawning.

Indeed, this somewhat informal association appears to be long‐standing. Carskadon (1993) in a study of EDS in normal subjects found that yawning was highly characteristic of sleepiness, as well as in clinical settings where an early American Sleep Disorders Association report on the use of the Multiple Sleep Latency Test (MSLT: Carskadon et al. 1986) recognised that observable indicators such as yawning, reduced activity, eyelid and head drooping and lapses in attention may be seen in patients who are sleepy (Thorpy 1992). Likewise, Baiardi et al. (2017) studied 60 patients using the Maintenance of Wakefulness Test (MWT: Mitler et al. 1982) [20 non‐sleepy (≥ 30′ sleep latency (SL)), 20 borderline (8′–30′ SL) and 20 sleepy (≤ 8′ SL)], and reported that ‘sleep‐fighting behaviours’ (i.e., yawns, facial expressions, body movements, scratching and face touching) were common, although eyelid closures were the most frequent signs in objectively sleepy subjects.

In response to my question about yawning in narcolepsy patients, Yves Dauvilliers (University of Montpellier, France) comments:

… (yawning) is a symptom to compensate, mostly to fight against sleepiness, and less a symptom or sign of excessive sleepiness … (yawning is) a highly variable symptom in hypersomnolence conditions (personal communication, December 18, 2024).

The same appears to be true of OSA and sleep‐related movement disorders (SRMD). Kosmas et al. (2012) reported that excessive yawning was not an inevitable characteristic of daytime sleepiness due to OSA. Rather absence of yawning bouts was thought to predict the presence of OSA. These authors suggested that this might be because OSA and yawning, sharing a thermoregulatory action but in opposite directions (heating/cooling respectively), may seldom coexist. Catli et al. (2015) reported that yawn frequency was moderately positively correlated with both EDS and ESS scores, but that it was also negatively moderately correlated with SaO_2_, leading to the suggestion that OSA physicians may use enquiry about yawning to help them predict the sleepiness of their patient. Kosmas et al. and Catli et al. therefore agree, albeit for different reasons, that yawning should be part of medical history‐taking from sleepy subjects. In the SRMD literature, periodic limb movement disorder (PLMD) and restless legs syndrome (RLS) excessive yawning is generally thought to reflect a common disturbance of dopaminergic neurotransmission (Leonhardt et al. 1999; Salas et al. 2010). Yawning is not mentioned in the International RLS Study Group Scale (Walters et al. 2003).

## 3 The Relevance of Yawning to Insomnia Disorder

Typically, in medicine and therapeutics, we look for symptoms and signs of a disorder and for foci and mechanisms of action of a treatment that lean towards *specificity*. In these respects, yawning appears disappointing. Although yawning may reflect a clinical condition, or even a sleep disorder, or an unwanted effect of a therapeutic or recreational drug, especially when its expression is excessive and functionally intrusive, the phenomenon is commonplace and non‐specific.

### 3.1 Why Might Yawning Be of Interest to Insomnia Disorder?

Where insomnia is concerned, there has been even less research conducted than in other sleep disorders. People with insomnia do yawn, but their rate of yawning may not be dissimilar (15–23 yawns per day) to good sleepers (Reid et al. 2004). Moreover, difficulty staying awake, a common precursor to yawning, appears to be the least endorsed feature of daytime complaint in DSM‐5‐defined insomnia, compared with lack of energy, low mood, poor concentration, impaired ability to get through work and poorer relationship functioning (Espie et al. 2012). Also, my own clinical experience would testify to the fact that few insomnia patients complain either about yawning too much or not enough. Furthermore, insomnia has been regarded as a disorder of *hyperarousal* (Riemann et al. 2010) or at least of a *failure to downregulate arousal* (C. A. Espie 2002, 2023). One might reasonably conclude, therefore, that yawning is largely irrelevant to insomnia.

However, an alternative perspective would be that it is precisely *because* yawning meets *sensitivity* criteria, being a truly ubiquitous phenomenon, and *because* it is likely a universal component of good sleep's normal *stimulus control paradigm* (C. A. Espie 2025, 59–71), that harnessing yawning as part of a novel behavioural therapy to address *faulty conditioned arousal* in insomnia may be worthwhile.

### 3.2 Yawning is Integral to Sleep Stimulus Control

As already demonstrated, yawning appears to serve important functions in physiology and social behaviour (Figure 2) with natural peaks preceding bedtime and after morning rising (Figure 3a,b). Box 1 illustrates the part that yawning may play in drawing attention to drowsiness and in initiating and supporting actions taken in favour of retiring to bed. According to the Psychobiological Inhibition Model and the Attention–Intention–Effort Pathway, which incorporate ‘adaptive conditioning’ as a core component, normal wake–sleep (and sleep–wake) transitions reflect the convergence of exogenous (environmental) and endogenous (internal) ‘setting conditions’ for sleep (C. A. Espie 2002, 2023; Espie et al. 2006). It is, therefore, plausible that yawning serves as a primary form of endogenous biofeedback communication (a symptom) to the individual, as well as an observable social communication (a sign) to others.

BOX 1 **Yawning plays a signalling role in the psychology of normal sleep.**

You begin to experience *endogenous* cues that are associated with physiological and mental de‐arousal (e.g., feeling sleepy, yawning, difficulty concentrating), and these seem to be associated with *exogenous* cues in the environment and in your behaviour (e.g., finishing your cup of tea, switching off the television, checking the time, locking the door).

You do not really know what triggers what. Is it the fact you looked at the clock that led you to yawn or vice versa? Or perhaps the closing credits for the programme you were watching are now on the screen, and this is what acted as a signal? You do not really know, but it does not strike you as even worth considering because this convergence of events is typical.

It is simply time for bed, you draw the threads together into a gestalt, and you may literally say to your partner what has become self‐evident to you: ‘Sorry, I'm struggling to stay awake; I need to get to my bed’.

*Source*: From C. A. Espie (2025, 60), reproduced with kind permission.

**FIGURE 4:**
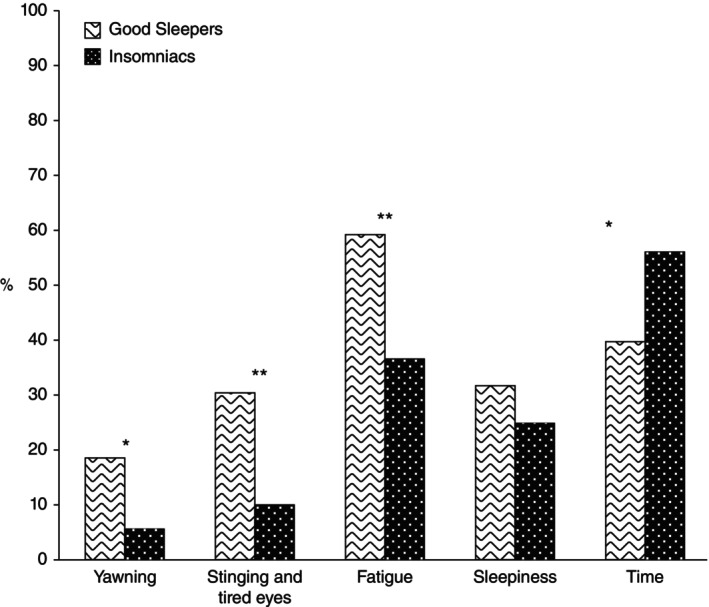
Percentages of good sleepers and Insomniacs (people with insomnia) reporting different sleep‐readiness signals in response to the question ‘*which are the signals you usually rely on to decide the best suitable time to sleep*?’ (*p < 0.05, **p < 0.01). From Giganti et al. (2016, 664), reproduced with kind permission.

Indeed, in instruction #1 of stimulus control therapy (SCT: i.e., ‘lie down intending to go to sleep only when you are sleepy’: Bootzin 1972, 395) clinicians are encouraged to ask their patients to observe the point at which they recognise that they are sleepy, rather than simply fatigued, for example, ‘Does he or she yawn or rub his or her eyes? Does his or her head begin to droop?’ (as explained in Bootzin and Epstein 2000, 176–177; see also Bootzin and Epstein 2011). Normal yawning then is explicitly cited in SCT as a marker to drive action (go to bed), consistent with Provine, Hamernik, and Curchack's (1987) suggestion that yawning is a paralinguistic signal for drowsiness. The need to emphasise such discrimination for adaptive sleep‐related behaviour is evident because people with insomnia may rely on what clock time it is to decide when to lie down with the intention of sleeping compared with good sleepers' greater reliance on yawning, stinging and tired eyes and fatigue (Figure 4: Giganti et al. 2016).

It is important to understand that the adult good sleeper is not good *at* sleeping any more than the baby who is a great sleeper is great *at* sleeping (cf. C. A. Espie 2025, 61). We are all born immature. There is an inherent drive towards structure and regularity of functions, but this requires exposure to the environment and consistent behavioural inputs to optimise development. So, for example, infants are born with the innate capability to speak, walk, use tools, take the perspective of others and so on, but they learn to do these things largely without awareness, a process called implicit learning (Meltzoff et al. 2009; Frensch and Rünger 2003). To support children's learning, adults make connections between new situations and familiar ones, focus attention, structure their experiences and organise the information they receive, while helping them also to develop strategies for intentional learning and problem‐solving (Darling‐Hammond et al. 2020). The same may be said for sleep patterns.

Sleep and circadian regularity first emerges in utero (Mirmiran et al. 2003) and in the first months of life becomes more measurable using core body temperature, levels of hormone secretion and the expression of clock genes (e.g., Joseph et al. 2015). There follows a maturational drive towards consolidated night‐time sleep. However, equilibrium is also facilitated by adaptive so‐called ‘sleep ecology’—actions taken by parents to support sleep initiation and resumption of sleep (e.g., nursing, rocking, holding, patting, singing and letting cry) as well as situational factors (e.g., sleep positioning, sleep arrangements [in crib/parent bed/elsewhere] and the presence/absence of a parent) (Sadeh et al. 2009; Mindell et al. 2010). It is in this context that yawning serves as one of the foremost signs that parents observe to interpret their child's level of arousal and readiness for sleep, and to use as a key ingredient in their infant sleep training. Popular children's books also refer to yawning, such as the Gruffalo's Child by Julia Donaldson (2004), ‘The Gruffalo sleeps on the cave floor. The Gruffalo's child yawns. The Gruffalo's child rubs her eye, then sits down sleepily against her dad’. Many similar materials help the parent–child dyad to share an understanding of sleep and its processes.

Crucially, when it becomes necessary to have clinically effective treatment of bedtime problems and night wakings in infants and young children, the most proven interventions largely optimise these same behavioural and situational factors that serve as determinants of healthy normal sleep (Mindell et al. 2006; Park et al. 2022). Finally, it is fascinating to note that even 3‐ to 8‐month‐old infants can themselves discriminate between yawning and unfamiliar mouth movements, which is well in advance of the development of contagious yawning (Tsurumi et al. 2019). This suggests that the neural mechanisms underlying yawning perception are in place early in life, reinforcing the behavioural and biofeedback utility of yawning.

It seems clear then that yawning may act as a specific discriminant stimulus within SCT, whether in parent behavioural sleep training or as part of CBT for adults. In keeping with Bootzin's protocol, I have always emphasised that feeling ‘*sleepy tired*’ is different from fatigue in that the former reflects an internal state of sleep readiness evidenced by lapses in concentration, yawning and periodic eye closure (e.g., C. A. Espie 1991, 158–159). Table 2 summarises this advice, and stresses that evidence of yawning is indicative of a struggle to remain awake, unlike other more circumstantial reasons to go to bed (C. A. Espie 2025). Importantly, the implementation of the return to bed component of SCT instruction #4 should also involve awareness of sleep readiness: ‘once again people with insomnia must attend to internal cues of sleepiness … when they begin to feel sleepy they should return to bed … (to) capture this sleepy moment and don't allow it to progress into a night‐time nap in the easy chair’ (Bootzin and Epstein 2000, 178).

**TABLE 2:** When should I go to bed? Going to bed when you feel ‘sleepy‐tired’, not for other reasons.

Poor reasons for deciding to go to bed (when you have insomnia)	Feeling ‘sleepy tired’: The best reasons for deciding to go to bed
It's late, it's past my bedtime	I'm struggling to stay awake
I've finished watching the TV	I'm yawning
There's nothing else to do	I've already nodded off
My partner is going to bed	I've lost track of what I was doing
I need to get up early tomorrow	Other people have noticed that I'm sleepy
I need to catch up on lost sleep	My eyes are itchy and I'm rubbing them
I slept really badly last night	My muscles ache and I need to stretch

*Note*: From C. A. Espie 2025, 196, reproduced with kind permission.

### 3.3 The Conditioned Yawn Reflex as Therapy (CYRaT) Offers a Novel Therapeutic for Insomnia Disorder

Behavioural sleep interventions, whether in children (see above) or adults (e.g., Edinger et al. 2021; Riemann et al. 2023 re SCT, sleep restriction therapy) are widely regarded as the most effective elements of the CBTx approach. My explanation for this is that strict behavioural management enables healthy sleep–wake functioning to (re)‐emerge when the setting conditions for sleep engagement and maintenance become adaptive and self‐sustaining. In brief, the most active ingredient in the CBTx formulary for insomnia is sleep itself (C. A. Espie 2025).

**FIGURE 5:**
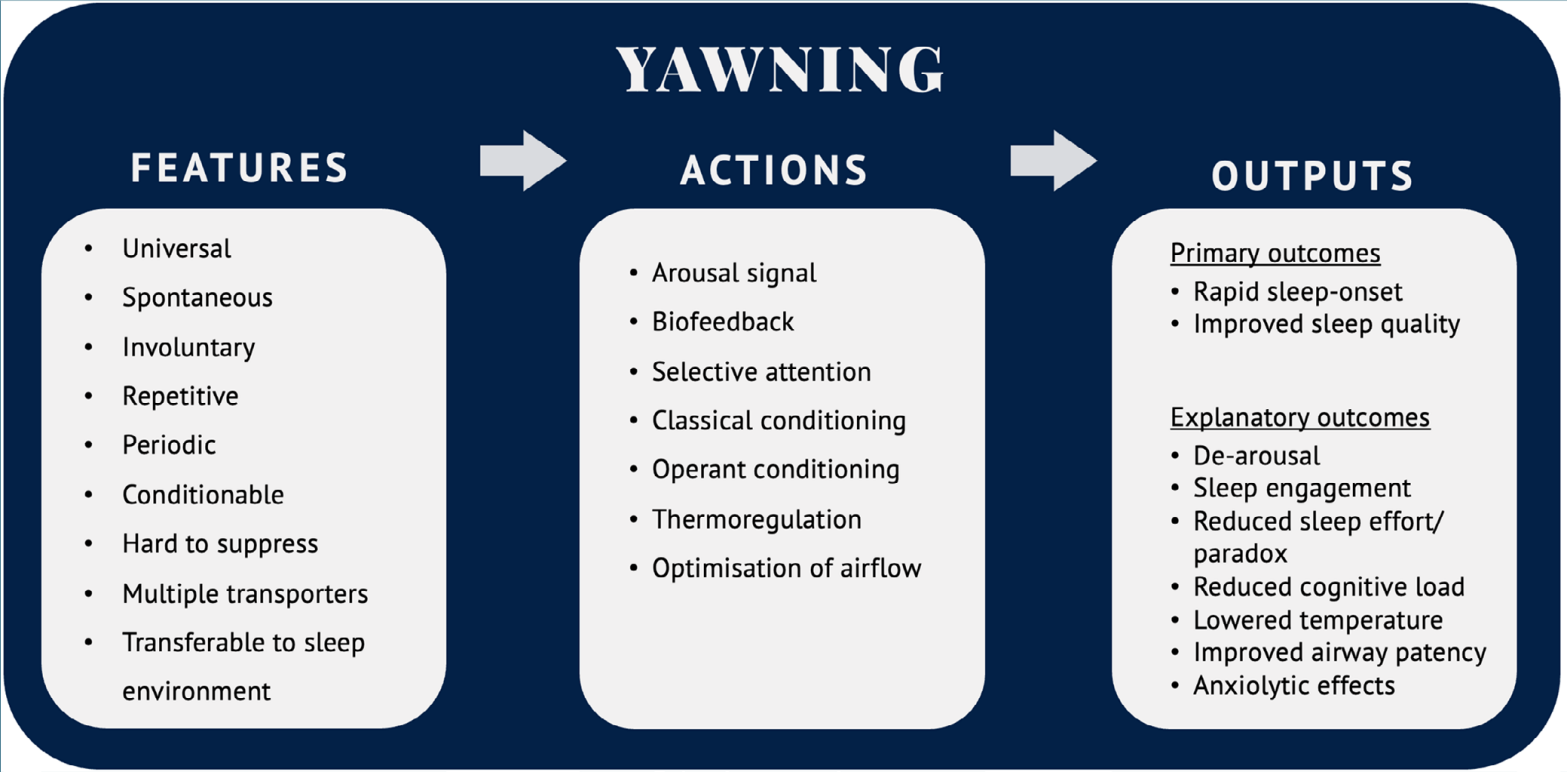
The suitability of yawning as a novel therapeutic for insomnia disorder.

Bearing in mind, therefore, the primary importance of nature's drives, processes and conserved features it may be wise to examine yawning more closely to see if there are additional ways that it may be utilised therapeutically. In Figure 5, and echoing earlier text and Figure 2, I suggest that yawning may be highly relevant to the behavioural management of insomnia for three reasons.

First, yawning has very distinctive *features*. In the introductory section, it was established that yawning has generalised, if not entirely universal, salience, that it is a reflexive SAP occurring spontaneously with a periodicity of recurrence, and importantly for our purposes here is both readily conditioned (contagion) and hard to suppress. In the usual course of events, contagious yawning may be passively incidental rather than actively purposeful. Nevertheless, the fact that yawn responses may be expected when a stimulus is presented paves the way for triggering a yawn, and thereby its further involuntary but potentially beneficial yawn sequences. Conditionability is favourable because yawning may be prompted by visual and/or auditory stimuli (as it were, standard social contagion), or by mere inner reflection upon yawning and by self‐instruction. These latter methods suggest that yawning may be readily activated in the bed and bedroom environment when a person is unable to sleep and may remain valid for people with ASD who are less susceptible to social contagion but who otherwise yawn similarly to other people.

Second, in terms of *actions*, these yawning features raise the possibility that triggering a repetitive yawn response in bed may signal and promote sleepiness in people with insomnia who often feel that they cannot get to sleep or cannot get back to sleep. The literature indicates that yawning is reliably related to present state arousal, at least as a cue, but perhaps also as a direct influence on sleep–wake transitions through the modulation of physiological arousal. Yawning therefore may offer biofeedback to the person with insomnia that their level of arousal is depleted rather than being sustained or enhanced. In turn, this would challenge their typical attention bias towards sleeplessness and unhelpful engagement in intentional and effortful strategies to overcome insomnia and drive selective attention to their involuntary drowsiness, and challenge dysfunctional thoughts concerned with the need to control sleep. Most importantly, CYRaT could capitalise on both the natural SAP of the yawn and therapeutic cycles of classical (associative) and operant conditioning (reinforcement), within and across nights, that might consolidate the ‘bed‐sleep connection’ (C. A. Espie 2021, 211) that is centrally important to SCT (Bootzin and Epstein 2000). Therapeutic yawning may also have sleep‐related benefits in terms of optimising airflow and brain cooling while in bed.

Third, the *outputs* or potential benefits of CYRaT are presented in the final column of Figure 4. It is hypothesised that the above adaptive conditioning paradigm would promote the important clinical outcomes of rapid sleep‐onset, both on retiring to bed and in the context of nocturnal awakenings, and improved sleep quality. Clearly much experimental work requires to be undertaken, followed by clinical trials, with suitably designed control conditions, but the hypothesis and proposed explanatory mechanisms are testable. For example, behaviour state changes may be measured in terms of physiological arousal (cortical, autonomic), cognitive–emotional factors (rating scales, attention bias experiments) and by temperature gradients and respiratory air flow. There is also the possibility that yawning has an anxiolytic effect, and/or a soporific effect because it is a repetitive event. Laboratory studies might examine the processes of conditioning, and another line of experimental work might utilise applied behaviour analysis of time series data using single case experimental designs to explore the treatment process (Barlow et al. 2009). Randomised trials would require a control condition with procedural similarities (e.g., sighing), and testing against established insomnia treatment like SCT would be appropriate if preliminary studies of CYRaT suggest clinical traction. Notably, and as already reviewed earlier in this article, there are established systems for identifying and recording yawn events themselves.

### 3.4 How Might a CYRaT Intervention for Insomnia Be Developed?

**FIGURE 6:**
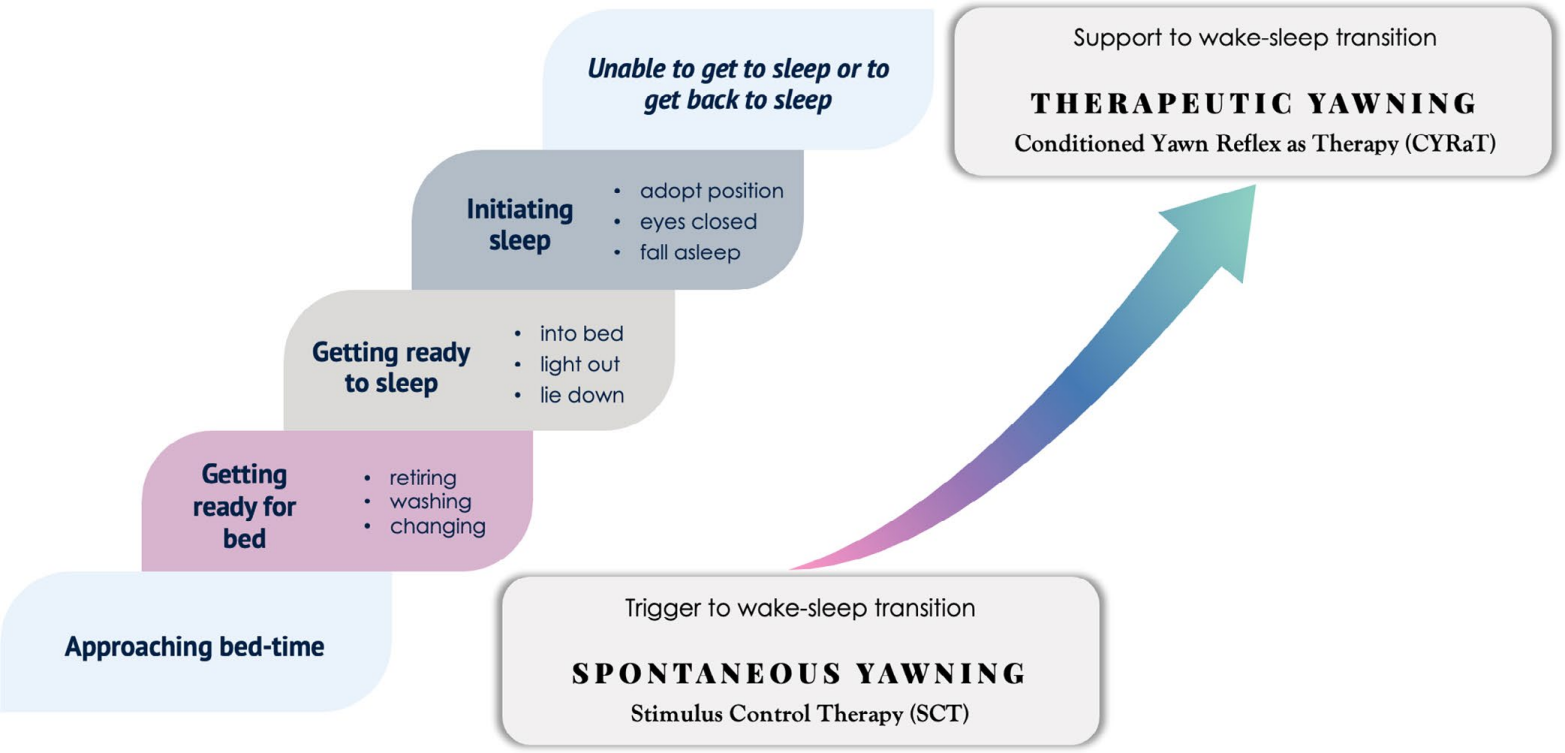
The salience of yawning in the steps leading up to sleep and to insomnia; from SCT to CYRaT.

**FIGURE 7:**
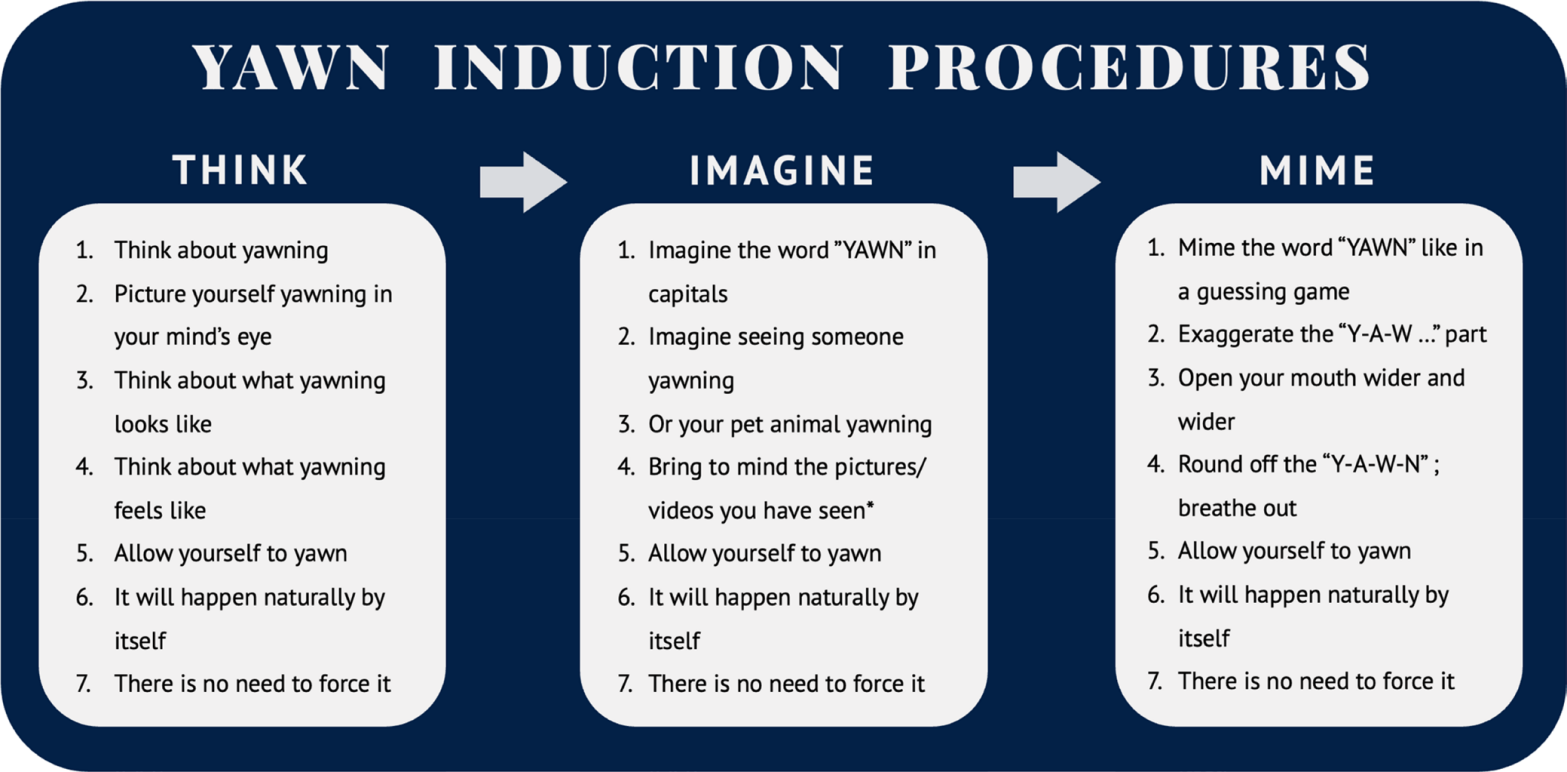
Potential yawn induction procedures for CYRaT that are self‐administered and directly applicable to the bedroom environment. *Refers to prior familiarisation training trials.

It is beyond the scope of this article to develop and instrument a definitive CYRaT intervention. That will need to be the product of careful iteration and testing. Nevertheless, a general framework can be offered. Figure 6 provides a useful starting point. The graphic illustrates several stages in the evening approach to sleep. We will subsequently turn to some specific examples of how CYRaT may be administered (Figure 7).

First, however, in Figure 6 it is proposed that yawning should be more explicitly recognised within our extant SCT protocols as a cardinal symptom and sign of sleepiness that should be (a) observed, (b) monitored, (c) evaluated and (d) welcomed and responded to by the patient as a reliable source of evidence for an imminent wake–sleep transition. The framing should be educational and engaging, so that patients can understand what yawning is and why we yawn, based upon the content of the early part of this article, distilled into readily accessible chunks of information. Emphasis should be placed upon differentiating sleepiness from fatigue, and that yawning may be an indicator of sleep readiness rather than just tiredness or boredom. Of course, not all yawning means that we need to head immediately to bed, but it should raise the question ‘why am I yawning at this time?’ A sleep diary adaptation may be helpful to record the timing and frequency of yawns, or a behavioural experiment might be conducted to test a hypothesis about an individual's natural sleep–wake pattern. With these latter points in mind, yawning and stretching are also common in the morning, associated with wakening and sleep inertia, and as noted earlier there may be chronotypical patterns that can be appraised. From a therapeutic standpoint we should help the patient to understand that sleepiness is an expression of a building sleep drive and that the opening of the circadian gate through which sleep may enter will likely be evidenced by bouts of spontaneous yawning. In this respect yawning can be explained as a ‘call to action’ in relation to their sleep readiness.

Second, as can be seen in Figure 6, yawning may continue to serve as biofeedback to the patient that they are on the right course of action in getting ready for bed and for sleep. Alternatively, it could be that yawning has served its purpose in behavioural activation towards bed and may not (need to) accompany them on this journey. These are empirical matters, and we need research to properly understand the role that yawning might play here. Nevertheless, most people become progressively sleepier as bedtime approaches (Figure 3a) so we should help patients understand and self‐monitor other symptoms and signs as well as yawning per se (e.g., rubbing eyes, losing concentration). Sleepiness symptoms may be experienced as pleasant and therefore intrinsically reinforcing, but we should also congratulate our patients for listening to their bodies and for making adaptive sleep preparation. We can help them develop reinforcing self‐statements about their sleep plan, to counter any doubts or dysfunctional thoughts that might interfere. Such cognitive elements may be important because in implementing what is for them a new sleep regime (based upon sleep‐readiness signals), it is likely that it will run counter to the more time‐ or situation‐based determinants of decision‐making, or ‘safety behaviours’ (Ree and Harvey 2004) that they have been used to (see Table 2 and Figure 4). The patient should be encouraged to see the continuity in the steps associated with getting ready for bed, getting ready to sleep and then initiating sleep. Analogous to implicit learning, as discussed earlier, the goal is to establish a pattern of behaviour that simply flows. There is innate sleep drive, we just need to go with the tide. Once in bed, the patient may or may not yawn. Again, these are empirical questions, and we need to gather the data, but my expectation would be that it is not uncommon for lying down and closing one's eyes to be associated with the last yawns of the evening.

Third, we have the situation where the insomnia patient is unable to get to sleep in the first place or to return to sleep following a wakeful episode during the night. It is in these situations where therapeutic yawning might be initiated. As above, psychoeducation will be useful to explain what yawns are, but in CYRaT a particular emphasis should be placed upon the contagious aspects of yawning and on how yawning is hard to suppress once it starts. Most likely, patients will know of this phenomenon and will be able to discuss examples of situations and experiences that they have had or that they have observed. As in therapy more generally, not least in paradoxical intention for insomnia, humour can play a part in de‐escalating perceived threat and helping patients take an alternative perspective (C. A. Espie 2025, chapters 10 and 11). People may also yawn at unexpected times or even in circumstances where it seems inappropriate. This too can be part of the discussion, but the main thrust of conversation should be the association of yawning with sleepiness and the struggle to remain awake. You might use Figure 6 to introduce yawning as a topic and to assist the patient in adopting a dependence upon sleep as the critical therapeutic ingredient that will help solve the insomnia dilemma. Explain that yawning is something that nature has done well and that it is nature's provision to guide us towards sleep through simple biofeedback. Explain that more than that, much of the animal kingdom responds contagiously to yawning, so we can apply the natural force of yawning to help us recreate that sense of sleepiness at times when we struggle with insomnia.

Finally, I have suggested three CYRaT techniques that might be implemented without the requirement for any stimuli in the bedroom. I trust that the content of Figure 7 is self‐explanatory. *Thinking*, *imagining*, and *miming* yawns all appear to be feasible and transportable to people with insomnia lying wakeful in bed. Each pathway has several key instructions, and all are followed by encouragement to permit yawning to emerge. Note that in relation to imagining yawns, I suggest that there may be some benefit in conducting a series of prior learning trials using either standardised or personalised audio–visual materials, which can then be recalled in situ without the stimuli present. However yawning is triggered, it seems likely that the conditioned yawns would persist in an involuntary reflexive manner, exhibiting a periodicity and intensity that doubtless would vary from person to person. It is hypothesised that CYRaT yawning would then deliver against the sleep‐onset and sleep quality outputs and explanatory factors proposed in Figure 5.

Of course, all of this remains entirely speculative, but hopefully intriguing enough to whet the appetite for some dedicated research attention.

## 4 Conclusion

Some years ago, the sleep scientist Jim Horne wondered why psychologists had not taken more interest in yawning, despite it being a universal human behaviour (Horne 2018). As mentioned in the introduction to this article, I had felt the same for some time, and this is my rather belated attempt to stimulate professionals working in the clinical psychology/behavioural sleep medicine domain to consider the potential of yawning as a therapeutic tool.

The possibility that a more explicit focus upon yawning as a cardinal symptom and sign, associated with the first principles of sleep‐related stimulus control therapy, is worthy of our attention as researchers and clinicians. In addition, the potential for manipulation of this ubiquitous, naturally occurring and adaptive, pre‐sleep phenomenon, which may be an integral part of the translation to sleep in normal subjects, to yield an entirely novel treatment intervention is an exciting one. It is exciting both in its simplicity and in its universal applicability to people with insomnia disorders. The well‐established responsivity of yawning responses to social and behavioural stimuli, on an apparently unselective basis, supports this contention. I hope that developing more refined protocols for the administration and testing of the Conditioned Yawn Response as Therapy (CYRaT) may be both feasible and fruitful. Time will tell, but the journey is likely to be intriguing.

## Author Contributions

**Colin A. Espie:** conceptualization, funding acquisition, writing – original draft, methodology.

## Conflicts of Interest

Colin A. Espie reports research support from the National Institute for Health and Care Research‐Health Technology Assessment (NIHR‐HTA; UK), the Medical Research Council, and The Wellcome Trust, and receiving payments from book publishing and lecture fees. He also reports being a Co‐Founder of and a shareholder in Big Health Ltd. (the developer of Sleepio). All outside the present work.

## Data Availability

Data sharing not applicable to this article as no datasets were generated or analysed during the current study.
